# Content and Composition of Branched-Chain Fatty Acids in Bovine Milk Are Affected by Lactation Stage and Breed of Dairy Cow

**DOI:** 10.1371/journal.pone.0150386

**Published:** 2016-03-01

**Authors:** Melissa L. Bainbridge, Laura M. Cersosimo, André-Denis G. Wright, Jana Kraft

**Affiliations:** 1 University of Vermont, Department of Animal and Veterinary Sciences, Burlington, Vermont, United States of America; 2 University of Arizona, School of Animal and Comparative Biomedical Sciences, Tucson, Arizona, United States of America; Humboldt-University Berlin, GERMANY

## Abstract

Dairy products contain bioactive fatty acids (FA) and are a unique dietary source of an emerging class of bioactive FA, branched-chain fatty acids (BCFA). The objective of this study was to compare the content and profile of bioactive FA in milk, with emphasis on BCFA, among Holstein (HO), Jersey (JE), and first generation HO x JE crossbreeds (CB) across a lactation to better understand the impact of these factors on FA of interest to human health. Twenty-two primiparous cows (n = 7 HO, n = 7 CB, n = 8 JE) were followed across a lactation. All cows were fed a consistent total mixed ration (TMR) at a 70:30 forage to concentrate ratio. Time points were defined as 5 days in milk (DIM), 95 DIM, 185 DIM, and 275 DIM. HO and CB had a higher content of n-3 FA at 5 DIM than JE and a lower n-6:n-3 ratio. Time point had an effect on the n-6:n-3 ratio, with the lowest value observed at 5 DIM and the highest at 185 DIM. The content of vaccenic acid was highest at 5 DIM, yet rumenic acid was unaffected by time point or breed. Total odd and BCFA (OBCFA) were higher in JE than HO and CB at 185 and 275 DIM. Breed affected the content of individual BCFA. The content of *iso*-14:0 and *iso*-16:0 in milk was higher in JE than HO and CB from 95 to 275 DIM. Total OBCFA were affected by time point, with the highest content in milk at 275 DIM. In conclusion, HO and CB exhibited a higher content of several bioactive FA in milk than JE. Across a lactation the greatest content of bioactive FA in milk occurred at 5 DIM and OBCFA were highest at 275 DIM.

## Introduction

There is growing awareness of the physiological and metabolic health properties of bioactive fatty acids (FA) derived from milk and dairy products. Bioactive FA in dairy products, such as α-linolenic acid (ALA; 18:3 *c*9,*c*12,*c*15), conjugated linoleic acids (CLA), and vaccenic acid (VA; 18:1 *t*11), are typically present in low percentages in milk (<5%), but exert a significant biological impact on human health [1]. CLA have been shown to have anti-carcinogenic effects [2], and research suggests VA can reduce tumor growth and the risk of cardiovascular diseases (CVD) [3], while ALA has demonstrated protective effects against inflammation [4], neurological disorders [5], and CVD [6]. This is supported by several observational studies and diet-intervention trials that associated milk consumption with a lower risk for CVD and type 2 diabetes [6–10]. Much of the research over the past decades has focused on maximizing these specific bioactive FA in milk, however, other unique bioactive lipids and FA have rarely been considered. For example, branched-chain FA (BCFA) are an emerging class of bioactive FA that exert cytotoxic effects on breast cancer cells [11], reduce the incidence of necrotizing enterocolitis in newborns [12], have anti-tumor effects on lymphomas [13], and improve pancreatic β-cell function [14]. Because BCFA originate from the cell membranes of rumen bacteria, dairy and meat products from ruminants are a unique source of these FA. This allows for BCFA to be used as biomarkers of dairy intake in humans and also as biomarkers of rumen function in cattle [15].

The content and composition of bioactive FA in milk fat is not constant, but varies markedly [16]. Composition of bioactive FA can be modified through several factors, such as animal genetics, environment, lactation stage, and diet [17]. Moreover, the content and profile of BCFA in milk fat varies depending on the activity and composition of the microbial populations (*i*.*e*., bacteria and protozoa) in the rumen, which are highly responsive to factors like diet [15,18] and host genetics (*i*.*e*., breed) [19]. Craninix *et al*. [20] examined the effect of lactation stage on milk odd and BCFA (OBCFA) in twenty primiparous and multiparous Holstein (HO) dairy cows throughout the first forty weeks of lactation. They determined that the content of *iso*-14:0, *anteiso*-15:0, and 15:0 increased with lactation stage, whereas 17:0 decreased from early to late-lactation. The composition of the diet was not consistent (*e*.*g*., adjustment of concentrate) and thus, caution must be taken when interpreting these results.

HO and Jersey (JE) dairy cattle are the two most prominent breeds in the U.S. [21]. HO cows are known for their high milk production, while JE cows are known for having a high fat and protein content in their milk [22]. Many dairy producers cross these two breeds to maintain elevated milk components and high milk production. Research shows that HO exhibit a greater content of CLA in milk fat when compared to other breeds, such as JE and Brown Swiss dairy cattle [23,24]. Palladino *et al*. [25] examined the FA profile of milk in HO and JE on a pasture-based diet and showed HO to have a lower content of saturated FA (SFA).

To the authors’ knowledge, no studies have quantified the effect of breed on the content and composition of BCFA in milk fat. The objectives of this study were to compare milk bioactive FA (with emphasis on BCFA), milk production, and milk components at four time points (5, 95, 185, and 275 DIM) between primiparous HO, JE, and first generation HO x JE crossbreeds (CB) fed the same diet across the lactation.

## Materials and Methods

### Experimental Design

All procedures using animals were approved by the University of Vermont (UVM) Institutional Animal Care and Use Committee under protocol # 14–034. Twenty-two primiparous cows of three breeds, HO (n = 7), JE (n = 8), and first generation CB (n = 7) were followed across a lactation. All cows calved within a two-month period and were co-housed at the UVM Paul Miller Research Facility from May of 2013 to May of 2014. Prior to calving, cows were fed a pre-partum total mixed ration (TMR; 70:30 forage to concentrate ratio; Table 1). Within 24 hours post-partum, cows were switched to a TMR formulated for lactating cows (70:30 forage to concentrate ratio; Table 1). Cows were fed twice daily, at 0600h and 1500h, for *ad libitum* intake (5–10% refusals) and had continuous access to water. Milking occurred twice daily at 0600h and 1700h and milk weights were recorded at each milking.

**Table 1:** Ingredient and chemical composition (mean ± standard deviation) of the pre-partum and post-partum diets.

	Diet	
	Pre-partum	Post-partum
%DM	36.8 ± 1.2	40.8 ± 1.1
Ingredient, TMR, % DM		
Corn silage	51.2	52.3
Haylage	8.3	15.9
Hay	13.3	—
Concentrate^a^	27.2	31.8
Chemical composition, % DM		
aNDFom^b^	35.0 ± 2.1	27.8 ± 1.1
CP (N x 6.25)^c^	14.1 ± 0.3	15.2 ± 0.7
NFC^d^	31.9 ± 4.8	39.7 ± 2.2
NE_L_^e^ Mcal/kg	1.44 ± 0.04	1.56 ± 0.02
Total fatty acids, % DM	1.97 ± 0.22	2.35 ± 0.07
Fatty acid composition (mg/g DM)		
16:0	4.18 ± 0.50	3.94 ± 0.21
18:0	0.62 ± 0.05	0.58 ± 0.04
18:1 *c*9	4.16 ± 0.81	4.99 ± 0.25
18:2 *c*9,*c*12	7.08 ± 1.22	9.64 ± 0.22
18:3 *c*9,*c*12,*c*15	2.07 ± 0.25	2.70 ± 0.28
Ʃ other^f^	1.62 ± 0.05	1.67 ± 0.05
Total SFA^g^	5.65 ± 0.53	5.25 ± 0.29
Total MUFA^h^	4.89 ± 0.92	5.87 ± 0.28
Total PUFA^i^	9.19 ± 1.04	12.40 ± 0.32
Total n-3 FA	2.07 ± 0.25	2.70 ± 0.28
Total n-6 FA	7.12 ± 1.21	9.70 ± 0.22

^*a*^The concentrate of the pre-partum diet contained (on DM basis): 16.1% ground soybean hulls, 16.9% Pasturechlor, 12.5% Soychlor, 15.7% canola meal, 18.8% amino max, 12.5% soybean meal, 4.4% calcium carbonate, 1.3% magnesium sulfate, 0.5% magnesium oxide, 0.5% sodium chloride, 0.7% trace minerals and vitamins, and 0.1% rumensin**®**. The concentrate of the post-partum diet contained (on DM basis): 16.4% soybean meal, 10.9% canola meal, 24.6% corn grain, 19.1% citrus pulp, 16.4% amino max, 5.5% PGI amino enhancer, 2.2% sodium sesquinate, 2.5% calcium carbonate, 1.2% sodium chloride, 0.43% trace minerals and vitamins, 0.05% zinc methionine, 0.65% magnesium oxide, 0.02% rumensin**®**.

^*b*^aNDFom = Ash-corrected neutral detergent fiber.

^*c*^CP = Crude protein.

^*d*^NFC = Non-fiber carbohydrate = 100 - (NDF + CP + ether extract + ash).

^*e*^NE_L_: Net energy lactation.

^*f*^Ʃ other: 12:0; 14:0; 15:0; 16:1 *t*9; 16:1 *c*9; 17:0; 18:1 *t*9; 18:1 *c*11; 20:0; 18:3 *c*6,*c*9,*c*12; 20:1 *c*8; 21:0; 20:2 *c*11,*c*14; 22:0; 22:1 *c*13; 20:4 *c*5,*c*8,*c*11,*c*14; 23:0; 24:0; 24:1 *c*15.

^*g*^SFA = Saturated fatty acids.

^*h*^MUFA = Monounsaturated fatty acids.

^*i*^PUFA = Polyunsaturated fatty acids.

### Data and Sample Collection

At the first time point, milk weights and samples were taken from 2 to 7 DIM (defined as time point 5 DIM) and composited by weight for each individual day. Cows were not sampled until colostrum excretion ceased. Every 90 days (after 5 DIM) samples were taken on days -2 to 2 relative to the sample time point and the five days of milk samples were composited by weight; these time points were denoted as 95, 185, and 275 DIM. An aliquot of each milk sample was preserved in 2-bromo-2-nitropropane-1,3-diol (Bronopol) and analyzed by mid-infrared spectroscopy for fat, crude protein, and organic solids by Lancaster Dairy Herd Improvement Association (Manheim, PA). A second aliquot was centrifuged at 3,434 x *g* for 30 minutes at 8°C and the cream layer was collected and stored at -20°C for FA analysis. Feed samples were taken three times per week, composited per time point, dried in a forced air oven at 65°C for 48 hours, and analyzed by Cumberland Valley Analytical Services (Hagerstown, MD) for chemical composition.

### Milk and Forage FA Analysis

FA composition of feed and milk samples was determined as described by Bainbridge *et al*. [26]. The content (g/kg) of individual FA in milk was calculated as follows, assuming milk fat to be 93.3% of FA [27]: *Total milk FA yield* (*g*/*d*) = [*Milk fat yield* (*kg*/*d*) *x* 1000] *x* 0.933

FAyield(g/d)=TotalmilkFAyield(g/d)x[FAproportion((g/100g))/100]

ContentofFA(g/kgmilk)=FAyield((g/d))/Milkyield(kg/d)

The content (mg) of individual FA in a serving of whole milk (3.25% milk fat; 244g [28]) was calculated as follows: *Total FA per serving* (*mg*) = 7.93*g fat*/*serving x* 0.933 *x* 1000

FAperserving(mg)=TotalFAperserving(mg)x[FAproportion(g/100g)/100]

### Statistical Analysis

Data were analyzed by repeated measures ANOVA using the PROC MIXED procedure in SAS 9.4 (SAS Institute, Cary, NC). Data were not obtained from one Jersey cow at 5 DIM due to illness, thus, data for that time point were considered missing in the model. The statistical model included the random effect of cow, fixed effect of breed, fixed effect of time point, the interaction of breed and time point, and residual error. The Kenward-Roger approximation was used for computing the denominator degrees of freedom for the tests of fixed effects resulting from the model. Least-squares (LS) means were generated using the LSMEANS/DIFF option to display the results. Data were adjusted for multiple comparisons using Bonferroni’s method. Significance was declared at *P*<0.05 and trends at 0.05≤*P*<0.10. *P*-values listed in the text refer to the main effects of either breed or lactation stage. Standard errors (SE) presented are averaged over all LS means. The small sample size in this study may have led to insufficient data to detect minor effects.

## Results

### Animal Production Parameters

#### Lactation Stage Differences

Overall, cows produced the most milk at 95 DIM (26.0 kg/d) and the least at 5 and 275 DIM (19.9 and 20.0 kg/d, respectively; *P*<0.001). Crude protein and organic solids yield was highest at 95 and 185 DIM (0.87 and 0.83 kg protein/d; 1.52 and 1.31 kg organic solids/d, respectively; *P*<0.001). Fat production differed in response to lactation stage, with cows producing less fat at 5 DIM than at all other time points (0.78, 1.15, 1.07, and 0.98 kg/d for 5, 95, 185, and 275 DIM, respectively; *P*<0.001). The significant interaction between breed and stage of lactation for milk fat and crude protein percentage (*P*<0.001) prohibits the comparison of main effects, but individual breeds across the lactation were considered. HO milk fat percentage did not vary across the lactation. In contrast, fat percentage in CB was higher at 275 DIM than at 5 DIM (Table 2) and the percentage of milk fat in JE increased at each time point until 185 DIM. HO had a higher percentage of milk crude protein at 5 DIM when compared to the subsequent time points. Milk crude protein percentage was highest in CB at 5 DIM and at 275 DIM and lowest at 95 DIM. In JE, milk crude protein percentage was highest at 185 and 275 DIM and lowest at 95 DIM.

**Table 2:** Milk yield and components of three breeds of dairy cow^a^ over four time points; 5 days in milk (DIM), 95 DIM, 185 DIM, 275 DIM.

	Time Point												SE	*P* value		
	5 DIM			95 DIM			185 DIM			275 DIM				B^*d*^	T^*e*^	B x T^*f*^
	HO	JE	CB	HO	JE	CB	HO	JE	CB	HO	JE	CB				
**Milk yield (kg/day)**	22.81	17.31	19.49	30.07	21.77	26.24	27.27	19.16	22.19	23.28	16.38	20.22	1.30	***	***	NS
**3.5% FCM**^*b*^	25.57	17.93	20.27	32.95	27.13	29.67	30.92	25.93	24.63	26.69	23.04	24.02	1.71	*	***	NS
**ECM**^*c*^	26.05	18.66	20.84	32.48	26.62	28.98	30.54	25.79	24.53	26.35	22.75	23.95	1.62	*	***	NS
**Milk components (kg/day)**																
**Fat**	0.97	0.65	0.73	1.23	1.09	1.13	1.18	1.09	0.93	1.03	0.98	0.94	0.07	NS	***	NS
**Crude protein**	0.88	0.67	0.73	0.98	0.78	0.84	0.93	0.79	0.77	0.80	0.68	0.75	0.05	*	***	NS
**Organic solids**	1.27	0.94	1.09	1.77	1.25	1.56	1.56	1.07	1.29	1.33	0.91	1.16	0.07	***	***	NS
**Milk components (%)**																
**Fat**	4.24	3.79	3.82	4.07	4.96	4.29	4.30	5.66	4.21	4.40	5.97	4.69	0.22	**	***	***
**Crude protein**	3.86	3.88	3.72	3.26	3.56	3.21	3.41	4.14	3.47	3.43	4.15	3.72	0.08	***	***	***
**Organic solids**	5.55	5.42	5.60	5.87	5.73	5.93	5.71	5.60	5.80	5.72	5.57	5.71	0.05	**	***	NS

^*a*^Least-squares (LS) means are based on n = 7 Holstein (HO), n = 8 Jersey (JE), and n = 7 HO x JE crossbreeds (CB).

^*b*^3.5% Fat-corrected milk = [0.4324 x milk yield (kg/d)] + [16.216 x fat yield (kg/d)].

^*c*^Energy-corrected milk = [12.82 x fat yield (kg/d)] + [7.13 x protein yield (kg/d)] + [0.323 x milk yield (kg/d)].

^*d*^Breed effect.

^*e*^Time point effect.

^*f*^Breed x time point interaction.

*P<0.05

**P<0.01

***P<0.001, NS = Not significant.

#### Breed Differences

Milk yield, 3.5% fat-corrected milk (FCM), and energy-corrected milk (ECM) differed by breed (Table 2); HO cows exhibited the highest milk yield across the lactation (25.9 kg/d), followed by CB (22.0 kg/d), and then JE (18.7 kg/d; *P*<0.001). FCM and ECM were higher in HO than JE and CB. Crude protein production differed between breeds; protein yield was overall highest in HO (0.90 *vs*. 0.73 and 0.77 kg/d for HO, JE, and CB, respectively; *P* = 0.01).

### Milk FA Composition

#### Lactations Stage Differences

The complete FA profile of milk, in g/100g FA, is presented in S1 Table. The milk FA profile was affected by lactation stage, with only two of the forty reported FA being unaffected by lactation stage (Tables 3 and 4). *De novo* synthesized FA (<16 carbon atoms) were lowest at 5 DIM then increased at each subsequent time point (7.61, 11.36, 12.60, and 13.32 g/kg milk for 5, 95, 185, and 275 DIM, respectively; *P*<0.001; Table 3). Conversely, preformed FA (>16 carbon atoms) were higher at 5 DIM (18.40 g/kg milk) when compared to the rest of lactation (14.43, 14.35, and 14.87 g/kg milk for 95, 185, and 275 DIM, respectively; *P*<0.001). Stage of lactation affected SFA; cows had the lowest content of SFA at 5 DIM, increasing at each subsequent time point (22.70, 29.62, 31.99, and 34.17 g/kg milk for 5, 95, 185, and 275 DIM, respectively; *P*<0.001).

**Table 3:** Content^a^ (g/kg milk) of major fatty acids in milk from three breeds of dairy cow over four time points; 5 days in milk (DIM), 95 DIM, 185 DIM, 275 DIM.

	Time Point													*P* Value		
	5 DIM			95 DIM			185 DIM			275 DIM			SE			
Fatty acid	HO	JE	CB	HO	JE	CB	HO	JE	CB	HO	JE	CB		B^*g*^	T^*h*^	B x T^*i*^
**4:0**	1.95	1.86	1.67	1.11	1.50	1.24	1.19	1.62	1.18	1.22	1.70	1.27	0.08	***	***	*
**6:0**	0.77	0.70	0.69	0.70	1.04	0.81	0.79	1.14	0.80	0.79	1.17	0.85	0.05	***	***	***
**8:0**	0.32	0.30	0.31	0.41	0.63	0.48	0.48	0.71	0.48	0.47	0.72	0.52	0.03	***	***	***
**10:0**	0.58	0.54	0.57	0.97	1.57	1.16	1.15	1.75	1.19	1.15	1.77	1.29	0.08	***	***	***
**12:0**	0.61	0.54	0.62	1.18	1.92	1.42	1.45	2.22	1.50	1.48	2.26	1.66	0.10	***	***	***
**14:0**	3.45	2.96	2.99	4.19	5.70	4.65	4.85	6.54	4.80	5.09	6.97	5.50	0.25	***	***	***
**14:1 *c*9**	0.14	0.09	0.12	0.30	0.34	0.35	0.39	0.51	0.42	0.42	0.52	0.49	0.03	NS	***	**
**16:0**	10.41	8.90	9.51	12.57	16.32	13.76	14.00	19.09	13.81	14.52	20.42	15.75	0.85	**	***	***
**16:1 *c*9**	0.64	0.45	0.56	0.61	0.58	0.63	0.61	0.83	0.66	0.68	0.89	0.80	0.07	NS	***	**
**18:0**	5.12	5.58	4.82	3.59	4.73	3.58	3.39	4.70	3.26	3.40	5.08	3.47	0.27	***	***	NS
**18:1 *t*9**	0.06	0.05	0.05	0.08	0.09	0.08	0.07	0.09	0.07	0.06	0.08	0.07	0.00	**	***	ƚ
**18:1 *t*10**	0.19	0.15	0.16	0.12	0.12	0.12	0.10	0.13	0.10	0.09	0.11	0.09	0.02	NS	***	NS
**18:1 *t*11**	0.57	0.47	0.63	0.39	0.44	0.37	0.34	0.41	0.29	0.35	0.44	0.31	0.04	NS	***	**
**18:1 *t*12**	0.10	0.08	0.09	0.13	0.14	0.12	0.11	0.14	0.11	0.10	0.13	0.10	0.01	*	***	*
**18:1 *t*13/*t*14**	3.90	3.86	3.32	0.16	0.24	0.18	0.16	0.23	0.16	0.17	0.22	0.15	0.28	NS	***	NS
**18:1 *c*9**	6.21	5.17	5.41	7.15	6.25	6.68	6.80	7.53	6.43	6.75	7.82	7.02	0.48	**	*	NS
**18:2 *c*9,*c*12**	0.59	0.65	0.58	0.60	0.64	0.60	0.61	0.72	0.59	0.56	0.71	0.61	0.05	*	NS	NS
**18:3 *c*9,*c*12,*c*15**	0.21	0.14	0.27	0.15	0.15	0.16	0.15	0.17	0.14	0.17	0.19	0.16	0.02	NS	**	*
**18:2 *c*9,*t*11**	0.21	0.14	0.21	0.19	0.16	0.18	0.17	0.17	0.15	0.18	0.18	0.17	0.02	NS	NS	NS
**20:5 *c*5,*c*8,*c*11,*c*14,*c*17**	0.03	0.02	0.04	0.01	0.01	0.02	0.01	0.01	0.01	0.01	0.01	0.01	0.00	ƚ	***	***
**22:5 *c*7,*c*10,*c*13,*c*16,*c*19**	0.06	0.04	0.06	0.03	0.03	0.04	0.03	0.02	0.03	0.02	0.02	0.02	0.00	NS	***	NS
**22:6 *c*4,*c*7,*c*10,*c*13,*c*16,*c*19**	0.01	0.01	0.01	0.00	0.00	0.00	0.00	0.00	0.00	0.00	0.00	0.00	0.00	*	***	**
**Unknown**	0.22	0.15	0.21	0.26	0.27	0.24	0.18	0.23	0.19	0.21	0.28	0.20	0.02	NS	***	***
***De novo***	8.22	7.28	7.32	9.61	13.59	10.88	11.09	15.57	11.13	11.40	16.20	12.37	0.61	***	***	***
**Mixed**	11.23	9.49	10.22	13.28	17.00	14.49	14.71	20.02	14.55	15.29	21.42	16.65	0.89	**	***	***
**Preformed**	19.47	18.20	17.54	14.44	14.92	13.93	13.66	16.39	12.98	13.59	17.10	13.92	1.07	ƚ	***	NS
**Total SFA**^*b*^	24.09	22.08	21.94	25.85	34.76	28.26	28.47	39.36	28.15	29.29	41.71	31.52	1.48	***	***	***
**Total MUFA**^*c*^	13.03	11.35	11.36	9.94	9.16	9.48	9.44	10.87	9.06	9.45	11.20	9.88	0.83	NS	**	NS
**Total PUFA**	1.44	1.26	1.48	1.32	1.35	1.34	1.30	1.49	1.23	1.31	1.54	1.32	0.10	NS	NS	NS
**Total 18:1 *trans***	1.01	0.83	1.02	0.82	0.91	0.80	0.72	0.89	0.67	0.69	0.86	0.66	0.06	NS	***	*
**Total n-6 FA**^*d*^	0.69	0.78	0.67	0.69	0.75	0.70	0.73	0.86	0.70	0.68	0.86	0.73	0.05	*	NS	NS
**Total n-3 FA**^*e*^	0.33	0.21	0.38	0.20	0.20	0.22	0.19	0.22	0.18	0.22	0.24	0.21	0.03	NS	***	**
**n-6:n-3 ratio**	2.19	3.75	1.98	3.51	3.69	3.23	3.78	4.03	3.79	3.15	3.74	3.47	0.19	***	***	*
**Total CLA**^*f*^	0.23	0.15	0.22	0.20	0.16	0.19	0.18	0.18	0.16	0.19	0.19	0.18	0.02	NS	NS	NS

^*a*^Least-sqaures (LS) means are based on n = 7 Holstein (HO), n = 8 Jersey (JE), and n = 7 HO x JE crossbreeds (CB).

^*b*^Total SFA: all saturated fatty acid (4:0 to 26:0).

^*c*^Total MUFA: all monounsaturated fatty acids (14:1 to 24:1).

^*d*^Total n-6 FA: all n-6 fatty acids; 18:2 *c*9,*c*12; 18:3 *c*6,*c*8,*c*12; 20:2 *c*11,*c*14; 20:3 *c*8,*c*11,*c*14; 20:4 *c*5,*c*8,*c*11,*c*14; and 22:4 *c*7,*c*10,*c*13,*c*16.

^*e*^Total n-3 FA: all n-3 fatty acids; 18:3 *c*9,*c*12,*c*15; 20:3 *c*11,*c*14,*c*17; 20:5 *c*5,*c*8,*c*11,*c*14,*c*17; 22:5 *c*7,*c*10,*c*13,*c*16,*c*19; and 22:6 *c*4,*c*7,*c*10,*c*13,*c*16,*c*19.

^*f*^Total CLA: all detected conjugated linoleic acid isomers: 18:2 *c*9,*t*11, 18:2 *t*11,*t*13, and 18:2 *t*7,*t*9/18:2 *t*10,*t*12.

^*g*^Breed effect.

^*h*^Time point effect.

^*i*^Breed x time point interaction.

ƚ 0.05≤*P*<0.10

*P<0.05

**P<0.01

***P<0.001, NS = Not significant

**Table 4:** Content^a^ (g/kg milk) of odd and branched-chain fatty acids (OBCFA) in milk from three breeds of dairy cow over four time points; 5 days in milk (DIM), 95 DIM, 185 DIM, 275 DIM.

	Time Point													*P* Value		
	5 DIM			95 DIM			185 DIM			275 DIM			SE			
Fatty acid	HO	JE	CB	HO	JE	CB	HO	JE	CB	HO	JE	CB		B^*f*^	T^*g*^	B x T^*h*^
**5:0**	0.02	0.01	0.01	0.02	0.01	0.02	0.03	0.04	0.03	0.02	0.03	0.02	0.00	NS	***	*
**7:0**	0.01	0.00	0.01	0.01	0.02	0.02	0.01	0.02	0.01	0.01	0.02	0.01	0.00	NS	***	*
**9:0**	0.01	0.00	0.01	0.02	0.02	0.02	0.02	0.03	0.02	0.02	0.02	0.01	0.00	*	***	NS
**11:0**	0.03	0.02	0.03	0.09	0.12	0.10	0.11	0.16	0.12	0.12	0.17	0.13	0.01	***	***	**
**13:0**	0.03	0.02	0.02	0.08	0.11	0.09	0.09	0.13	0.09	0.09	0.13	0.10	0.01	**	***	**
***iso*-13:0**	0.01	0.01	0.01	0.01	0.01	0.01	0.01	0.01	0.01	0.02	0.02	0.02	0.00	NS	***	NS
***anteiso-*13:0**	0.01	0.01	0.01	0.02	0.04	0.03	0.04	0.05	0.04	0.04	0.06	0.05	0.00	**	***	*
***iso*-14:0**	0.03	0.03	0.03	0.03	0.06	0.05	0.04	0.07	0.05	0.06	0.08	0.07	0.00	***	***	***
**15:0**	0.32	0.25	0.28	0.52	0.59	0.52	0.52	0.68	0.50	0.53	0.72	0.53	0.03	**	***	**
***iso-*15:0**	0.09	0.07	0.07	0.08	0.09	0.08	0.08	0.10	0.08	0.10	0.13	0.10	0.00	**	***	***
***anteiso-*15:0**	0.13	0.11	0.12	0.18	0.18	0.17	0.18	0.21	0.17	0.20	0.25	0.21	0.00	ƚ	***	**
***iso-*16:0**	0.11	0.09	0.09	0.08	0.13	0.10	0.11	0.18	0.11	0.14	0.21	0.17	0.01	***	***	***
**17:0**	0.38	0.31	0.32	0.29	0.32	0.29	0.28	0.37	0.28	0.29	0.39	0.31	0.02	*	*	**
***iso-*17:0**	0.15	0.12	0.14	0.13	0.12	0.12	0.11	0.13	0.11	0.12	0.14	0.13	0.01	NS	**	*
***anteiso*-17:0**	0.17	0.15	0.13	0.04	0.05	0.04	0.06	0.06	0.04	0.03	0.04	0.04	0.01	NS	***	NS
**17:1 *c*9**	0.18	0.12	0.13	0.09	0.07	0.08	0.08	0.09	0.07	0.08	0.09	0.08	0.01	NS	***	*
***iso*-18:0**	0.00	0.00	0.00	0.01	0.01	0.00	0.00	0.00	0.01	0.01	0.01	0.00	0.00	NS	*	NS
**Total OBCFA**	2.28	1.82	1.92	2.19	2.50	2.24	2.32	2.96	2.22	2.42	3.21	2.55	0.11	**	***	***
**Total BCFA**^*b*^	0.70	0.58	0.59	0.57	0.69	0.61	0.65	0.82	0.62	0.71	0.94	0.78	0.03	**	***	***
**Total OCFA**^*c*^	1.59	1.24	1.33	1.62	1.81	1.63	1.67	2.14	1.60	1.71	2.27	1.77	0.09	**	***	***
**Total *iso* BCFA**^*d*^	0.39	0.32	0.34	0.33	0.42	0.37	0.36	0.50	0.37	0.43	0.59	0.48	0.02	**	***	***
**Total *anteiso* BCFA**^*e*^	0.31	0.26	0.25	0.24	0.27	0.24	0.29	0.32	0.26	0.28	0.35	0.30	0.02	*	***	**

^*a*^Least-sqaures (LS) means are based on n = 7 Holstein (HO), n = 8 Jersey (JE), and n = 7 HO x JE crossbreeds (CB).

^*b*^Total BCFA: all branched-chain fatty acids (*iso-*13:0 to *iso-*18:0 and *anteiso-*13:0 to *anteiso*-17:0).

^*c*^Total OCFA: all odd-chain fatty acids (5:0 to 17:0).

^*d*^Total *iso* BCFA: all *iso* branched-chain fatty acids (*iso-*13:0 to *iso-*18:0).

^*e*^Total *anteiso* BCFA: all *anteiso* branched-chain fatty acids (*anteiso-*13:0 to *anteiso*17:0).

^*f*^Breed effect.

^*g*^Time point effect.

^*h*^Breed x time point interaction.

ƚ 0.05≤*P*<0.10

*P<0.05

**P<0.01

***P<0.001, NS = Not significant.

Total OBCFA, BCFA, *iso*-14:0, 15:0, *iso*-15:0, *anteiso*-15:0, *iso*-16:0, 17:0, and *iso*-17:0 differed across the lactation, however, the significant interaction between breed and time point prohibits the examination of main effects (Table 4). The content of OBCFA and BCFA in milk fat from JE increased at each time point. CB had the highest content of OBCFA and BCFA in milk fat at 275 DIM. The content of OBCFA in HO did not differ across the lactation, although BCFA were lower at 95 DIM when compared to all other time points. The content of *iso*-14:0 in JE milk increased at each time point from 5 to 275 DIM. HO had the lowest content of *iso*-14:0 at 5 and 95 DIM and the highest content at 275 DIM. CB also had the lowest content of *iso*-14:0 at 5 DIM and highest at 275 DIM. HO and CB had a lower content of 15:0 in milk at 5 DIM when compared to the subsequent time points. Similarly, JE had the lowest content of 15:0 at 5 DIM and the highest at 185 and 275 DIM. The content of *iso*-15:0 in HO and CB was highest at 275 DIM. In JE, the content of *iso*-15:0 increased at each time point from 5 DIM. HO and CB had the lowest content of *anteiso*-15:0 at 5 DIM and highest at 275 DIM. Comparably, the content of *anteiso*-15:0 in JE was lowest at 5 DIM and increased at each subsequent time point. The content of *iso*-16:0 was highest in all breeds at 275 DIM. In JE, *iso*-16:0 increased at each time point, while HO had the lowest content of *iso*-16:0 at 95 DIM. Across the lactation, the content of 17:0 in milk from CB did not vary. HO had a higher content of 17:0 at 5 DIM, whereas JE had a lower content of 17:0 at 5 DIM when compared to 185 and 275 DIM. In JE cows the content of *iso*-17:0 was highest at 275 DIM, while in HO the content of *iso*-17:0 was highest at 5 DIM. Similar to HO, the content of *iso*-17:0 in milk of CB was higher at 5 DIM than at 95 and 185 DIM. In all breeds, the proportion of *anteiso-*17:0 was highest at 5 DIM and lowest at 95 and 275 DIM (0.15, 0.04, 0.06, and 0.04 g/kg milk for 5, 95, 185, and 275 DIM, respectively; *P*<0.001).

As a result of the significant interaction between breed and time point, total 18:1 *t*, VA, n-3 FA, n-6:n-3 ratio, ALA, and eicosapentaenoic acid (20:5 *c*5,*c*8,*c*11,*c*14,*c*17; EPA) will be compared across a lactation by individual breed (Table 3). The content of total 18:1 *t*, VA, and n-3 FA in milk fat from JE did not vary across the lactation. Contrary, HO and CB had a higher content of total 18:1 *t*, VA, and n-3 FA at 5 DIM when compared to the subsequent time points. ALA was the main driver of the increased n-3 FA, with HO and CB having a higher content of ALA at 5 DIM than all other time points, while the content of ALA in milk fat of JE did not differ across the lactation. EPA followed the same pattern, with HO and CB having a higher content of EPA in milk at 5 DIM and JE showing no change across the lactation. The n-6:n-3 ratio of JE milk did not differ across the lactation. Milk fat from HO and CB had a lower n-6:n-3 ratio at 5 DIM than the remainder of lactation. In all breeds, docosapentaenoic acid (22:5 *c*7,*c*10,*c*13,*c*16,*c*19) was higher at 5 DIM when compared to later time points (0.06, 0.03, 0.03, and 0.02 g/kg milk for 5, 95, 185 and 275 DIM, respectively; *P*<0.001). Docosahexaenoic acid (22:6 *c*4,*c*7,*c*10,*c*13,*c*16,*c*19) was only present in contents greater than 0.01 g/kg milk at 5 DIM. Total PUFA, n-6 FA, and total CLA were unaffected by lactation stage.

#### Breed Differences

Across the lactation there were marked differences in the milk FA profile between breeds (Tables 3 and 4). The significant interaction between breed and lactation stage for *de novo* FA and SFA is due to the effect of breed being non-significant at 5 DIM, although, from 95 to 275 DIM, production of *de novo* FA was higher in JE than HO and CB (15.12 *vs*. 10.70 and 11.46 g/kg milk, respectively; *P*<0.001). Similarly, the proportion SFA was greater in JE than HO and CB from 95 to 275 DIM (38.61 *vs*. 27.87 and 29.31 g/kg milk, respectively; *P*<0.001).

JE had a higher content of total OBCFA in milk fat at 185 and 275 DIM than HO and CB (Table 4). At 275 DIM JE also had a higher content of total BCFA. The contents of *iso*-14:0 and *iso-*16:0 were higher in JE at 95 and 275 DIM. At 185 and 275 DIM 15:0, *iso*-15:0, and *anteiso*-15:0 were higher in JE than in CB and HO. Content of 17:0 in milk was higher in HO than in JE and CB at 5 DIM, yet by 275 DIM the content of 17:0 was highest in JE. Similarly, the content of *iso*-17:0 at 5 DIM was higher in HO than JE, while at 275 DIM the content of *iso*-17:0 was higher in JE than HO. MUFA, PUFA, CLA, total *iso* FA, total *anteiso* FA, *anteiso*-17:0, and 18:1 *t* were unaffected by breed. At 5 DIM, HO and CB had a higher content of n-3 FA than JE (Table 3). Overall, milk from JE had a greater content of n-6 FA than HO and CB (0.81 *vs*. 0.70 and 0.70 g/kg milk, respectively; *P*<0.05) resulting in higher n-6:n-3 ratio when compared to HO and CB at 5 DIM.

## Discussion

The main objective of this study was to compare bioactive milk FA, with emphasis on BCFA, between HO, JE, and CB fed the same diet across a lactation. The three breeds were chosen because of their high prevalence on U.S. dairy farms and primiparous cows were used to eliminate the effect of parity on FA content in milk. Diet is acknowledged as a major factor affecting the FA profile of milk fat [16]. The diet of the dairy cattle changed at parturition from a TMR formulated for pre-partum cows to a TMR formulated for lactating cows to meet the energy and nutrient demands of the animal, which is typical practice on dairy farms. The data at the 5 DIM time point are a reflection of the transition between the two diets, and thus, changes between this time point and other time points in lactation may reflect both the effect of diet and the effect of stage of lactation. The FA contents were reported in g/kg milk to show the impact of breed and lactation (*i*.*e*., production). These units also eliminate the difficulty in determining whether a change in the proportion (g/100g FA) of a FA occurred because of an actual change in the amount of that FA, or a change in the total amount of FA [29]. Results discussed from other studies were converted to g/kg milk for comparison when data were available, if data were unavailable (data presented in figures only, or production data not presented) then results were compared in g/100g FA. The authors would also like to note that the small sample size in this study may have resulted in insufficient power, thus some non-significant results may reach significance in a study with more observations.

### Animal Production Parameters

As expected, HO cows produced more 3.5% FCM and ECM than JE and CB, agreeing with research conducted at the herd level [30], and in both pasture and TMR systems [23]. The observation of higher milk fat and crude protein concentrations from 95 to 275 DIM in JE than HO and CB is consistent with previous research between HO and JE [23,31], while Palladino *et al*. [25] found CB to have an intermediate concentration of milk fat and crude protein that was different from both HO and JE. In addition, no difference between the fat yield of HO and JE was observed by Palladino *et al*. [25], however, they found fat yield to be higher in CB than JE, while no difference was observed in the current study.

### Milk OBCFA

**Fig 1:**
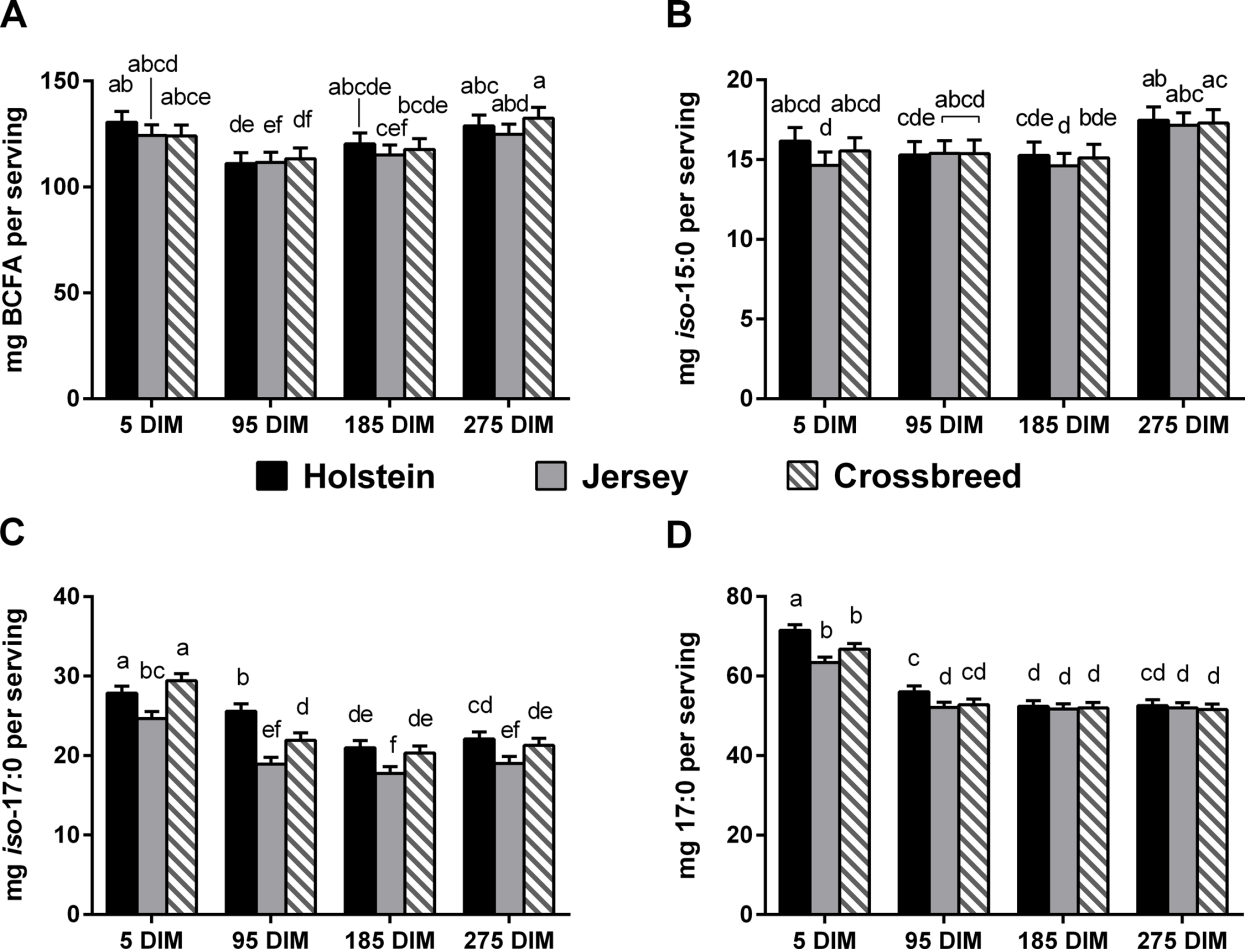
**Content of total branched-chain fatty acids (BCFA) [A], *iso-*15:0 [B], *iso-*17:0 [C], and 17:0 [D] per serving of whole milk (3.25% milk fat).** Means are presented as least-squares (LS) means (n = 7 Holstein (HO), n = 8 Jersey (JE), and n = 7 HO x JE crossbreeds (CB)) and standard error. LS means without a common letter differ significantly (*P*<0.05). DIM = Days in milk.

The predominant OBCFA detected in milk fat in this study were 15:0, 17:0, *anteiso*-15:0, *iso*-17:0, *iso*-16:0, *iso*-15:0, *anteiso*-17:0, and *iso*-14:0, which is in agreement with other research [18,32]. Ruminant products are a unique source of OBCFA in the human diet, as OBCFA are synthesized by rumen bacteria and protozoa [33]. Small amounts of 15:0 and 17:0 can also be synthesized *de novo* from propionate in the mammary gland and adipose tissue of ruminants [18,34]. In addition, research suggests some capacity for the mammary gland to elongate *iso*-15:0 and *anteiso*-15:0 to *iso*-17:0 and *anteiso*-17:0, respectively [35]. Hence, levels of 15:0 and 17:0 have been used as markers for dairy and ruminant fat intake in humans and to identify correlations between intake of ruminant products [36] and risk for disease, such as metabolic syndrome [37,38]. Palladino *et al*. [25] examined mid-lactation HO, JE, and CB, demonstrating the content of 15:0 to be higher in JE than HO and CB (0.56 *vs*. 0.45 and 0.52 g/kg milk) and no difference among breeds in the content of 17:0, agreeing with our findings during the mid-lactation time points (95 and 185 DIM). Craninx *et al*. [20] followed HO dairy cows over 40 weeks of lactation and observed the lowest proportion (g/100g FA) of 15:0 in milk during early-lactation, matching our observations in HO cows at 5 DIM. They also noted a higher proportion (g/100g FA) of 17:0 in milk fat of early-lactation cattle and attributed this to a higher content of 17:0 in adipose tissue [20]. Thus, mobilization of non-esterified FA from adipose tissue in early-lactation would increase the supply of this FA to the mammary gland. The FA profile of adipose tissue was not determined in the present study, although, plasma non-esterified FA (data not shown) and 17:0 in milk were higher at 5 DIM than all other time points. In the current study, the content of 17:0 was higher in a serving of whole milk from cows at 5 DIM when compared to all subsequent time points (Fig 1), while the content of 15:0 in a serving of whole milk was lowest at 5 DIM (57 *vs*. 98, 95, and 93 mg/serving; *P*<0.001). These results need to be taken into consideration when using these FA as biomarkers for ruminant product consumption in humans.

BCFA may function to lower the melting point of butter, creating a more desirable product as these FA generally function to maintain the fluidity of bacterial cell membranes [39], and *anteiso* BCFA in particular, have a low melting point relative to chain length [18,40]. BCFA have been proposed as a potential biomarker for rumen function [35,41]. Vlaeminck *et al*. [42] developed regression equations showing milk *iso*-14:0 and *iso*-15:0 to be positively related to rumen concentrations of acetate. The total BCFA content (g/kg milk and g/100g FA) was affected by stage of lactation, which agrees with research done by Craninx *et al*.[20]. The shift in the BCFA across the lactation, however, does not match the pattern of the present study. Craninx *et al*. [20] observed an increase in BCFA (g/100g FA) until week 12 of lactation (approx. 84 DIM) when the content plateaued. The present study demonstrated a higher proportion of BCFA (g/100g; S1 Table) at 5 DIM and at 275 DIM, with a lower proportion at 95 and 185 DIM. Discrepancies could be a result of the experimental design, such as the inclusion of multiparous cows, basal diet composition, or the adjustment of concentrate fed across the lactation according to the animal’s requirements by Craninx *et al*. [20].

**Fig 2:**
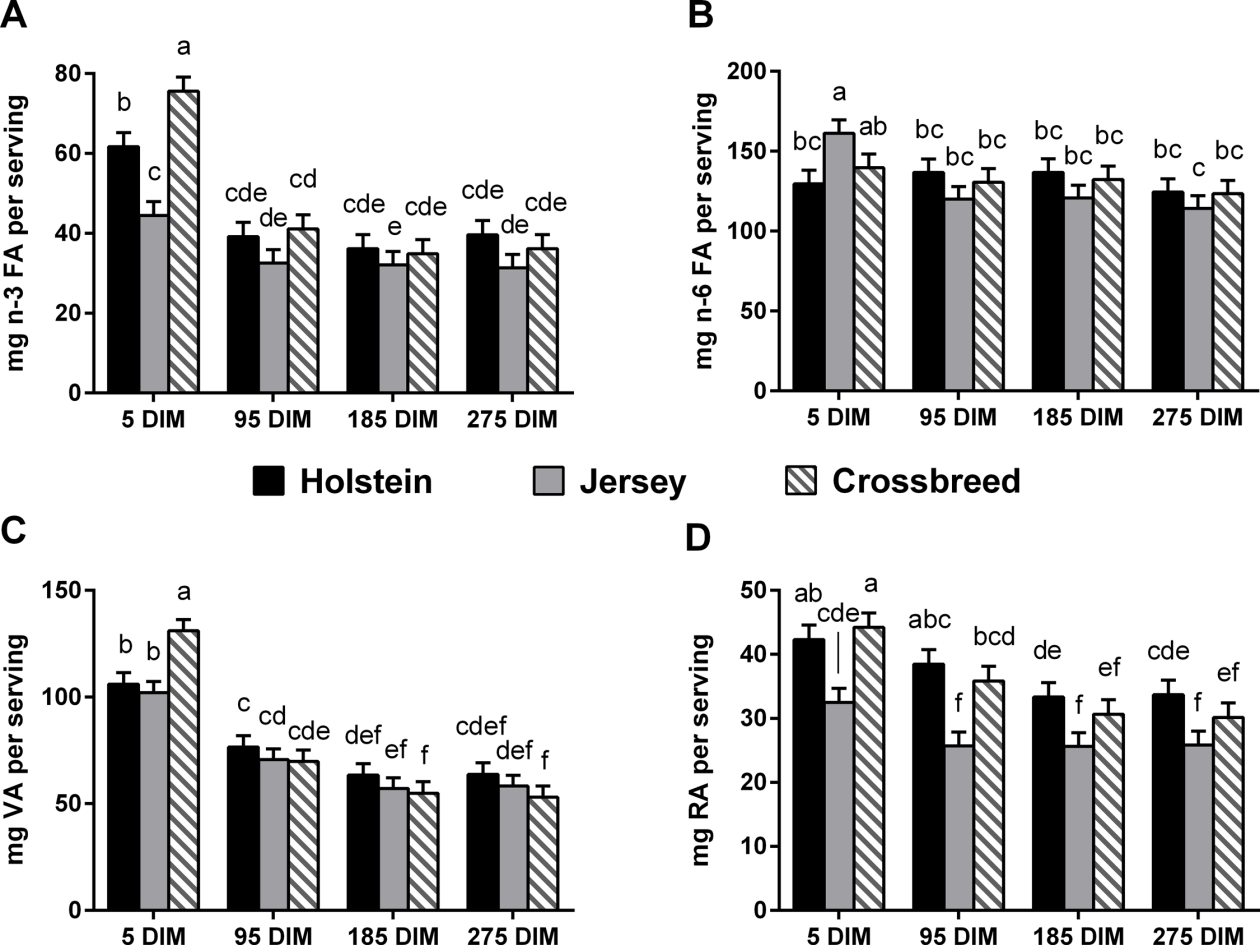
**Content of n-3 FA [A], n-6 FA [B], vaccenic acid (VA) [C], and rumenic acid (RA) [D] per serving of whole milk (3.25% milk fat).** Means are presented as least-squares (LS) means (n = 7 Holstein (HO), n = 8 Jersey (JE), and n = 7 HO x JE crossbreeds (CB)) and standard error. LS means without a common letter differ significantly (*P*<0.05). DIM = Days in milk.

Research demonstrates that BCFA possess anticarcinogenic properties [11,43]. Wongtangtintharn *et al*. [11] observed anti-tumor activity with *iso*-16:0, and the content of this FA was highest in a serving of whole milk from cows at 275 DIM (27.0 mg/serving). The inhibitory effect of *iso*-15:0 on several malignant tumor cell lines was tested by Yang *et al*. [43]. The results showed that *iso*-15:0 could inhibit tumor growth at a dosage of 35 mg/kg body weight in mice. A serving of whole milk in this study provided 16 mg of *iso*-15:0, thus, more research needs to be done to show if the average content of *iso*-15:0 in milk fat exhibits bioactivity in humans. Additionally, when examining the total content of BCFA known to have anticarcinogenic properties, the contribution of milk fat to the bioactive dose is more significant. The content of *iso*-15:0 in a single serving of whole milk was unaffected by breed, but was higher in a serving of whole milk from cows at 275 DIM when compared to the other time points (Fig 1). This is consistent with research done by Baumann *et al*. [44] who examined the OBCFA composition (mg/g) of milk fat from seven HO cows across five time points in lactation and found *iso*-15:0 to be highest from 120 to 310 DIM. *anteiso*-15:0 has been shown to have similar toxicity in tumor cells to *iso*-15:0 [11]. The content of this BCFA was overall higher per serving of whole milk from HO than from JE (32.7 *vs*. 28.8 mg/serving), and CB were not different from the other breeds (31.5 mg/serving, *P*<0.05). Despite the statistical significance, a 4 mg difference in BCFA content per serving is negligible for human nutrition. Similarly to *iso*-15:0, content of *anteiso*-15:0 in a serving of whole milk was highest in milk from cows at 275 DIM (35.3 mg/serving) and lowest in milk from cows at 5 DIM (24.3 mg/serving), agreeing with results from Baumann *et al*. [44]. Overall, a serving of whole milk from cows at 5 DIM provided the highest content of *iso*-17:0 (Fig 1), comparable to results by Baumann *et al*. [44] who observed the highest content of *iso*-17:0 in milk from HO cows at 5–15 DIM. HO consistently had a higher content of *iso*-17:0 per serving of whole milk than JE (Fig 1). BCFA are a substantial component of the gastrointestinal tract of newborns [45], and have been shown to prevent necrotizing enterocolitis in a neonatal rat model [12]. A serving of whole milk contains on average 121 mg of BCFA, but only 42 mg of total n-3 FA (Figs 1 and 2). This content is lower, but similar, to that observed by Ran-Ressler *et al*. [32] who examined the BCFA content of retail milk, approximating 158 mg BCFA/per serving of whole milk.

### Other Milk FA of Importance to Human Health

Individual SFA can have varied effects on disease outcomes. Palmitic acid (16:0) has been correlated with decreased insulin sensitivity [46] whereas, stearic acid (18:0) may have a protective effect against cardiovascular disease [47], and very-long-chain SFA (>22 carbon atoms) have been associated with a lower risk for diabetes [48]. Opposing effects on plasma lipids have been shown for myristic acid (14:0), depending on dietary intake, with a moderate intake leading to a higher plasma high-density lipoprotein level [49,50]. Thus, when considering the contributions of SFA in a food product to human health, not only the overall SFA content but also the composition of SFA needs to be considered. In accordance with other research, the content of total SFA in milk fat was higher in JE compared with HO, which is often associated with a greater *de novo* synthesis of FA in JE [51,52]. Myristic acid and palmitic acid were lowest at 5 DIM, then increased at each subsequent time point. Nantapo *et al*. [53] observed HO, JE, and CB in a pasture system at three stages of lactation, and also found myristic acid to be highest at late-lactation (291 DIM). However, unlike in the current study, they observed the lowest content of myristic acid at 135 and 174 DIM, not at 5 DIM. Palladino *et al*. [25] examined milk FA of mid-lactation (approx. 120 DIM) HO, JE, and CB on a pasture-based diet during June and July, demonstrating palmitic acid to be higher in JE and CB than HO (12.1 and 10.6 *vs*. 8.4 g/kg milk). Our research also showed the content of palmitic acid to be highest in JE, although it was lower in CB than JE, and more consistent with the content of palmitic acid in milk from HO. Overall, stearic acid was highest at 5 DIM. These results are similar to Kay *et al*. [54] who examined the milk FA profile of HO cows at 1, 8, and 16 weeks of lactation and observed a higher proportion of stearic acid during the first week of lactation when compared to week 16 (approximately 112 DIM). Stearic acid comprises 12–18% of total FA in adipose tissue of ruminants [55,56]. The mobilization of FA during early lactation to meet the energy demands of the animal [57] may have led to the observed higher content of this FA in milk at 5 DIM.

VA is the precursor to rumenic acid (RA; 18:2 *c*9,*t*11), the predominant CLA isomer in milk fat. VA reduces the risk for atherosclerosis [58], as well as contributes to the levels of RA in tissues by conversion via Δ-9 desaturase [59]. VA was highest in a serving of whole milk from cows at 5 DIM (Fig 2). Breed did not affect the content of VA, which agrees with data published by Pallidino *et al*. [25]. Notably, although differences were observed in the content of VA across the lactation, RA was not affected by lactation stage. Neither Pallidino *et al*. [25] nor Nantapo *et al*. [53] observed breed differences when comparing RA between HO, JE, and CB, agreeing with results presented in the current study. Yet, the content of RA in a serving of whole milk was affected by breed (Fig 2), with milk from HO providing approximately 10 mg more RA per serving than milk from JE. This demonstrates the importance in comparing results in equivalent units. Kelsey *et al*. [24] has also shown the proportion (g/100g FA) of CLA in milk fat to be higher in HO than JE. This could be due to lower Δ-9 desaturase activity in JE *vs*. HO cattle [60]. Both the 18:0 [18:1 *c*9/(18:0 + 18:1 *c*9)] and 18:1 *t*11 [18:2 *c*9,*t*11 /(18:1 *t*11 + 18:2 *c*9,*t*11)] Δ-9-desaturase indices were lower in JE than HO and CB, agreeing with research by Pallidino *et al*. [25] who observed lower desaturase indices in JE than HO dairy cattle.

Content of total n-3 FA and ALA in a single serving of whole milk was lower in JE than HO and CB at 5 DIM (Fig 2). At all other time points, the content of total n-3 FA and ALA in a serving of whole milk was not different between breeds. At 5 DIM, the content of n-3 FA in a serving of whole milk was highest for HO and CB breeds when compared to the subsequent time points. ALA content (g/kg milk) in milk decreased from 5 to 275 DIM, agreeing with results presented by Nantapo *et al*. [53]. The breed difference in the content (g/kg milk) of ALA at 5 DIM, however, was not observed by Nantapo *et al*.[53]. This may be due to the difference in sampling days (5 DIM in this study *vs*. 28 DIM [53]). The current recommendation of adequate intake of n-3 FA by the Institute of Medicine of the National Academies is 1.6 g/day for men and 1.1 g/day for women, or 0.6–1.2% of energy intake, with up to 10% of this value from EPA and DHA [61]. In the current study a serving of whole milk provides an average of 41.6 mg of n-3 FA and 130.5 mg n-6 FA. These values are consistent with the content of n-3 (40.7 mg) and n-6 (153.1 mg) FA in retail milk of conventional farms in northeast England [62]. Moreover, dairy cooperatives are moving towards paying farmers based on the content of bioactive FA in their milk (personal communication), thus, these data could be used by farmers to improve their profitability and the healthfulness of their product. The n-6:n-3 ratio was lower in HO and CB than JE at 5 DIM. Overall, this ratio was lowest in milk at 5 DIM, and highest in milk fat at 185 DIM, the ratio in milk fat was consistently at or below 4:1, which is considered to be the upper limit of the optimal ratio (1:1 to 4:1) [63].

## Conclusion

U.S. American nutrition guidelines state that most dairy product choices should be fat-free or reduced-fat, based on the potentially detrimental health effects of SFA [64]. Nonetheless, there is no conclusive evidence that whole milk consumption is harmful to human health [7]. The reduced intake of milk fat in response to these guidelines may result in consumers lacking in various bioactive FA provided by whole-fat dairy products. The profile and content of bioactive FA in milk varies with stage of lactation and breed. A serving of whole milk from HO cows has a higher content of RA, *anteiso-*15:0, *iso*-17:0, and n-3 FA (at 5 DIM) than milk from JE cows. Overall, milk from cows at 5 DIM had a higher content of n-3 FA, VA, CLA, *iso-*17:0, and 17:0. This research suggests that HO cows have a more desirable FA profile from a human health standpoint on a per serving basis, although the numeric difference in total bioactive FA (n-3, VA, CLA, and OBCFA) is modest (281 *vs*. 253 mg per serving for HO and JE cows, respectively). Regardless of breed, stage of lactation was the predominant factor affecting bioactive FA, with milk from cows at 5 DIM containing a higher amount of bioactive FA than cows at 275 DIM on a per serving basis (340 *vs*. 240 and 253 mg/serving for 5, 185, and 275 DIM, respectively). Additional research is needed to elucidate the mechanism of the shifts in the milk OBCFA to in order to develop dairy products with an enhanced profile.

## Supporting Information

S1 Table Fatty acid concentration^*a*^ (g/100g FA) in milk fat from three breeds of dairy cow over four time points; 5 days in milk (DIM), 95 DIM, 185 DIM, 275 DIM. ^*a*^Least-squares (LS) means are based on n = 7 Holstein (HO), n = 8 Jersey (JE), and n = 7 HO x JE crossbreeds (CB). ^*b*^Total SFA: all saturated fatty acid (4:0 to 26:0). ^*c*^Total MUFA: all monounsaturated fatty acids (14:1 to 24:1). ^*d*^Total n-6 FA: all n-6 fatty acids; 18:2 n-6, 18:3 n-6, 20:2 n-6, 20:3 n-6, 20:4 n-6, and 22:4 n-6. ^*e*^Total n-3 FA: all n-3 fatty acids; 18:3 n-3, 20:3 n-3, 20:5 n-3, 22:5 n-3, and 22:6 n-3. ^*f*^Total CLA: all detected conjugated linoleic acid isomers: 18:2 *c*9,*t*11, 18:2 *t*11,*t*13, and 18:2 *t*7,*t*9/18:2 *t*10,*t*12. ^*g*^Breed effect. ^*h*^Time point effect. ^*i*^Breed x time point interaction. ƚ 0.05≤*P*<0.10; *P<0.05; **P<0.01; ***P<0.001, NS = Not significant.
(DOCX)
