# Supplementary material for: Content and Composition of Branched-Chain Fatty Acids in Bovine Milk Are Affected by Lactation Stage and Breed of Dairy Cow
Source: PLoS One. 2016 Mar 1;11(3):e0150386. doi: 10.1371/journal.pone.0150386 (PMC4773160; doi:10.1371/journal.pone.0150386)
Supplement: S1 Table — aLeast-squares (LS) means are based on n = 7 Holstein (HO), n = 8 Jersey (JE), and n = 7 HO x JE crossbreeds (CB). bTotal SFA: all saturated fatty acid (4:0 to 26:0). cTotal MUFA: all monounsaturated fatty acids (14:1 to 24:1). dTotal n-6 FA: all n-6 fatty acids; 18:2 n-6, 18:3 n-6, 20:2 n-6, 20:3 n-6, 20:4 n-6, and 22:4 n-6. eTotal n-3 FA: all n-3 fatty acids; 18:3 n-3, 20:3 n-3, 20:5 n-3, 22:5 n-3, and 22:6 n-3. fTotal CLA: all detected conjugated linoleic acid isomers: 18:2 c9,t11, 18:2 t11,t13, and 18:2 t7,t9/18:2 t10,t12. gBreed effect. hTime point effect. iBreed x time point interaction. ƚ 0.05≤P<0.10; *P<0.05; **P<0.01; ***P<0.001, NS = Not significant. (DOCX) [file pone.0150386.s001.docx]

|  | Time Point | | | | | | | | | | | | | | | SE | *P* Value | | |
| --- | --- | --- | --- | --- | --- | --- | --- | --- | --- | --- | --- | --- | --- | --- | --- | --- | --- | --- | --- |
|  | 5 DIM | | |  | 95 DIM | | |  | 185 DIM | | |  | 275 DIM | | |  |  | | |
| Fatty acid | HO | JE | CB |  | HO | JE | CB |  | HO | JE | CB |  | HO | JE | CB |  | B*^g^* | T*^h^* | B x T*^i^* |
| 4:0 | 4.97 | 5.37 | 4.69 |  | 2.93 | 3.25 | 3.11 |  | 2.99 | 3.06 | 2.97 |  | 2.97 | 3.04 | 2.90 | 0.15 | NS | *** | NS |
| 5:0 | 0.04 | 0.03 | 0.03 |  | 0.04 | 0.03 | 0.04 |  | 0.07 | 0.08 | 0.07 |  | 0.04 | 0.05 | 0.05 | 0.01 | NS | *** | NS |
| 6:0 | 1.95 | 2.04 | 1.97 |  | 1.86 | 2.25 | 2.03 |  | 1.98 | 2.17 | 2.03 |  | 1.92 | 2.09 | 1.93 | 0.10 | ƚ | NS | NS |
| 7:0 | 0.02 | 0.01 | 0.02 |  | 0.04 | 0.04 | 0.04 |  | 0.03 | 0.04 | 0.03 |  | 0.03 | 0.03 | 0.02 | 0.00 | NS | *** | NS |
| 8:0 | 0.82 | 0.86 | 0.90 |  | 1.09 | 1.37 | 1.20 |  | 1.18 | 1.34 | 1.21 |  | 1.15 | 1.28 | 1.17 | 0.06 | ƚ | *** | NS |
| 9:0 | 0.02 | 0.01 | 0.02 |  | 0.05 | 0.05 | 0.05 |  | 0.05 | 0.05 | 0.04 |  | 0.04 | 0.04 | 0.03 | 0.00 | NS | *** | NS |
| 10:0 | 1.48 | 1.56 | 1.67 |  | 2.56 | 3.38 | 2.87 |  | 2.85 | 3.31 | 3.00 |  | 2.80 | 3.17 | 2.92 | 0.15 | * | *** | * |
| 11:0 | 0.08 | 0.07 | 0.08 |  | 0.23 | 0.26 | 0.25 |  | 0.28 | 0.31 | 0.30 |  | 0.28 | 0.30 | 0.29 | 0.01 | NS | *** | NS |
| 12:0 | 1.53 | 1.56 | 1.82 |  | 3.12 | 4.15 | 3.53 |  | 3.60 | 4.20 | 3.81 |  | 3.59 | 4.05 | 3.77 | 0.17 | * | *** | ** |
| *iso*-13:0 | 0.02 | 0.02 | 0.02 |  | 0.03 | 0.03 | 0.03 |  | 0.03 | 0.03 | 0.03 |  | 0.04 | 0.04 | 0.04 | 0.00 | NS | *** | NS |
| *anteiso*-13:0 | 0.02 | 0.02 | 0.02 |  | 0.06 | 0.08 | 0.08 |  | 0.09 | 0.10 | 0.10 |  | 0.10 | 0.11 | 0.11 | 0.01 | NS | *** | NS |
| 13:0 | 0.06 | 0.05 | 0.07 |  | 0.22 | 0.23 | 0.22 |  | 0.23 | 0.25 | 0.24 |  | 0.22 | 0.24 | 0.22 | 0.01 | NS | *** | NS |
| *iso*-14:0 | 0.09 | 0.10 | 0.08 |  | 0.07 | 0.13 | 0.12 |  | 0.11 | 0.13 | 0.12 |  | 0.13 | 0.15 | 0.15 | 0.01 | * | *** | ** |
| 14:0 | 8.78 | 8.56 | 8.57 |  | 11.07 | 12.31 | 11.64 |  | 12.08 | 12.35 | 12.20 |  | 12.36 | 12.48 | 12.57 | 0.40 | NS | *** | NS |
| *iso*-15:0 | 0.22 | 0.20 | 0.21 |  | 0.21 | 0.21 | 0.21 |  | 0.21 | 0.20 | 0.20 |  | 0.24 | 0.23 | 0.23 | 0.01 | NS | *** | NS |
| 14:1 *t*9 | 0.00 | 0.00 | 0.00 |  | 0.01 | 0.01 | 0.01 |  | 0.01 | 0.01 | 0.01 |  | 0.01 | 0.01 | 0.01 | 0.00 | NS | *** | NS |
| *anteiso-*15:0 | 0.33 | 0.31 | 0.34 |  | 0.47 | 0.40 | 0.43 |  | 0.46 | 0.40 | 0.45 |  | 0.50 | 0.44 | 0.49 | 0.02 | NS | *** | NS |
| 14:1 *c*9 | 0.35 | 0.24 | 0.34 |  | 0.78 | 0.74 | 0.86 |  | 0.96 | 0.96 | 1.07 |  | 1.00 | 0.93 | 1.13 | 0.04 | * | *** | NS |
| 15:0 | 0.81 | 0.72 | 0.80 |  | 1.39 | 1.28 | 1.29 |  | 1.30 | 1.28 | 1.27 |  | 1.29 | 1.29 | 1.20 | 0.06 | NS | *** | NS |
| *iso-*16:0 | 0.27 | 0.27 | 0.26 |  | 0.20 | 0.29 | 0.26 |  | 0.27 | 0.33 | 0.29 |  | 0.34 | 0.37 | 0.39 | 0.02 | NS | *** | * |
| 16:0 | 26.33 | 25.01 | 26.61 |  | 33.03 | 35.17 | 34.42 |  | 34.62 | 36.14 | 35.03 |  | 35.12 | 36.60 | 35.90 | 0.83 | NS | *** | NS |
| *iso-*17:0 | 0.38 | 0.33 | 0.40 |  | 0.35 | 0.26 | 0.30 |  | 0.28 | 0.24 | 0.27 |  | 0.30 | 0.26 | 0.29 | 0.01 | ** | *** | * |
| 16:1 *t*9 | 0.10 | 0.09 | 0.09 |  | 0.03 | 0.02 | 0.02 |  | 0.02 | 0.01 | 0.02 |  | 0.02 | 0.02 | 0.02 | 0.01 | NS | *** | NS |
| 16:1 isomer | 0.01 | 0.01 | 0.01 |  | 0.01 | 0.01 | 0.01 |  | 0.01 | 0.01 | 0.01 |  | 0.01 | 0.01 | 0.01 | 0.00 | NS | *** | NS |
| 16:1 *c*7 | 0.04 | 0.03 | 0.04 |  | 0.03 | 0.02 | 0.03 |  | 0.02 | 0.02 | 0.03 |  | 0.02 | 0.02 | 0.02 | 0.00 | * | *** | NS |
| 16:1 *c*8 | 0.20 | 0.20 | 0.20 |  | 0.16 | 0.11 | 0.15 |  | 0.14 | 0.11 | 0.13 |  | 0.14 | 0.11 | 0.13 | 0.01 | ** | *** | ƚ |
| *anteiso-*17:0 | 0.44 | 0.43 | 0.35 |  | 0.09 | 0.11 | 0.10 |  | 0.16 | 0.12 | 0.11 |  | 0.08 | 0.08 | 0.09 | 0.02 | NS | *** | NS |
| 16:1 *c*9 | 1.63 | 1.22 | 1.51 |  | 1.59 | 1.25 | 1.57 |  | 1.52 | 1.56 | 1.70 |  | 1.64 | 1.60 | 1.84 | 0.10 | NS | ** | * |
| 16:1 *c*10/*t*13 | 0.01 | 0.01 | 0.01 |  | 0.01 | 0.01 | 0.01 |  | 0.01 | 0.00 | 0.01 |  | 0.01 | 0.01 | 0.00 | 0.00 | NS | *** | NS |
| 16:1 *c*11 | 0.07 | 0.05 | 0.07 |  | 0.05 | 0.03 | 0.04 |  | 0.04 | 0.04 | 0.04 |  | 0.05 | 0.05 | 0.05 | 0.01 | NS | *** | NS |
| 17:0 | 0.97 | 0.86 | 0.90 |  | 0.76 | 0.70 | 0.71 |  | 0.71 | 0.70 | 0.70 |  | 0.71 | 0.70 | 0.70 | 0.02 | ƚ | *** | NS |
| *iso*-18:0 | 0.01 | 0.01 | 0.00 |  | 0.01 | 0.01 | 0.01 |  | 0.01 | 0.01 | 0.02 |  | 0.01 | 0.01 | 0.01 | 0.00 | NS | * | * |
| 17:1 *t*10 | 0.11 | 0.09 | 0.10 |  | 0.06 | 0.05 | 0.05 |  | 0.05 | 0.05 | 0.04 |  | 0.05 | 0.05 | 0.05 | 0.00 | ƚ | *** | NS |
| 17:1 *c*8 | 0.03 | 0.02 | 0.03 |  | 0.02 | 0.01 | 0.02 |  | 0.02 | 0.01 | 0.01 |  | 0.02 | 0.01 | 0.02 | 0.00 | *** | *** | NS |
| 17:1 *c*9 | 0.46 | 0.32 | 0.37 |  | 0.24 | 0.16 | 0.21 |  | 0.19 | 0.17 | 0.18 |  | 0.19 | 0.17 | 0.19 | 0.02 | ** | *** | * |
| 18:0 | 12.93 | 15.94 | 13.76 |  | 9.45 | 10.32 | 8.95 |  | 8.52 | 8.91 | 8.20 |  | 8.40 | 9.14 | 7.91 | 0.50 | * | *** | * |
| 18:1 *t*4 | 0.01 | 0.01 | 0.01 |  | 0.02 | 0.02 | 0.01 |  | 0.01 | 0.01 | 0.01 |  | 0.01 | 0.01 | 0.01 | 0.00 | NS | *** | NS |
| 18:1 *t*5 | 0.01 | 0.01 | 0.01 |  | 0.02 | 0.02 | 0.01 |  | 0.01 | 0.01 | 0.01 |  | 0.01 | 0.01 | 0.01 | 0.00 | NS | *** | NS |
| 18:1 *t*6-8 | 0.22 | 0.22 | 0.25 |  | 0.25 | 0.23 | 0.23 |  | 0.22 | 0.20 | 0.21 |  | 0.20 | 0.18 | 0.17 | 0.01 | NS | *** | NS |
| 18:1 *t*9 | 0.15 | 0.16 | 0.15 |  | 0.21 | 0.19 | 0.20 |  | 0.18 | 0.17 | 0.18 |  | 0.16 | 0.14 | 0.15 | 0.01 | NS | *** | NS |
| 18:1 *t*10 | 0.47 | 0.42 | 0.42 |  | 0.33 | 0.26 | 0.30 |  | 0.25 | 0.24 | 0.26 |  | 0.21 | 0.19 | 0.22 | 0.03 | NS | *** | NS |
| 18:1 *t*11 | 1.43 | 1.38 | 1.77 |  | 1.04 | 0.96 | 0.94 |  | 0.86 | 0.77 | 0.74 |  | 0.86 | 0.79 | 0.72 | 0.07 | NS | *** | ** |
| 18:1 *t*12 | 0.24 | 0.23 | 0.26 |  | 0.33 | 0.30 | 0.31 |  | 0.28 | 0.27 | 0.29 |  | 0.26 | 0.23 | 0.23 | 0.01 | NS | *** | NS |
| 18:1 *t*13-14 | 10.01 | 10.59 | 8.86 |  | 0.43 | 0.52 | 0.45 |  | 0.40 | 0.44 | 0.41 |  | 0.42 | 0.39 | 0.35 | 0.50 | NS | *** | NS |
| 18:1 *c*9 | 15.54 | 14.23 | 15.25 |  | 18.73 | 13.50 | 16.71 |  | 17.11 | 14.26 | 16.47 |  | 16.60 | 14.09 | 16.18 | 0.82 | ** | * | * |
| 18:1 *c*11 | 0.94 | 0.90 | 0.82 |  | 0.80 | 0.52 | 0.66 |  | 0.65 | 0.54 | 0.60 |  | 0.53 | 0.50 | 0.51 | 0.05 | * | *** | * |
| 18:1 *c*12 | 0.14 | 0.16 | 0.15 |  | 0.26 | 0.26 | 0.27 |  | 0.23 | 0.24 | 0.25 |  | 0.23 | 0.20 | 0.22 | 0.01 | NS | *** | NS |
| 18:1 *c*13 | 0.13 | 0.10 | 0.11 |  | 0.08 | 0.04 | 0.06 |  | 0.05 | 0.04 | 0.05 |  | 0.05 | 0.04 | 0.05 | 0.01 | NS | *** | NS |
| 18:1 *c*14/*t*16 | 0.28 | 0.26 | 0.30 |  | 0.30 | 0.29 | 0.29 |  | 0.26 | 0.25 | 0.25 |  | 0.26 | 0.24 | 0.23 | 0.01 | NS | *** | NS |
| 18:1 *c*15 | 0.17 | 0.16 | 0.14 |  | 0.09 | 0.08 | 0.09 |  | 0.08 | 0.06 | 0.07 |  | 0.08 | 0.06 | 0.07 | 0.01 | NS | *** | NS |
| 18:2 *t*10,*t*14 | 0.03 | 0.01 | 0.05 |  | 0.09 | 0.10 | 0.10 |  | 0.07 | 0.07 | 0.07 |  | 0.08 | 0.08 | 0.07 | 0.01 | NS | *** | NS |
| 18:2 *t*9,*t*12 | 0.03 | 0.01 | 0.03 |  | 0.00 | 0.00 | 0.01 |  | 0.00 | 0.00 | 0.00 |  | 0.00 | 0.00 | 0.00 | 0.00 | ** | *** | *** |
| 18:2 *c*9,*t*13/*t*8,*c*12 | 0.14 | 0.11 | 0.15 |  | 0.18 | 0.13 | 0.17 |  | 0.16 | 0.14 | 0.16 |  | 0.16 | 0.11 | 0.14 | 0.01 | * | *** | ƚ |
| cyclohexyl-11 11:0 | 0.07 | 0.06 | 0.08 |  | 0.15 | 0.14 | 0.16 |  | 0.17 | 0.13 | 0.17 |  | 0.17 | 0.13 | 0.15 | 0.01 | ** | *** | NS |
| 18:2 *c*9,*t*14 | 0.08 | 0.05 | 0.07 |  | 0.11 | 0.08 | 0.09 |  | 0.09 | 0.07 | 0.09 |  | 0.09 | 0.06 | 0.08 | 0.01 | ** | *** | NS |
| 18:1 *c*16 | 0.04 | 0.05 | 0.05 |  | 0.06 | 0.06 | 0.05 |  | 0.05 | 0.04 | 0.05 |  | 0.05 | 0.04 | 0.04 | 0.01 | NS | ** | NS |
| 18:2 *c*12,*t*16 | 0.00 | 0.00 | 0.00 |  | 0.02 | 0.02 | 0.02 |  | 0.02 | 0.02 | 0.02 |  | 0.02 | 0.02 | 0.02 | 0.00 | NS | *** | NS |
| 18:2 *t*9,*c*12 | 0.14 | 0.08 | 0.19 |  | 0.09 | 0.08 | 0.10 |  | 0.08 | 0.05 | 0.07 |  | 0.11 | 0.09 | 0.07 | 0.01 | ** | *** | *** |
| 18:2 *t*11,*c*15 | 0.03 | 0.02 | 0.03 |  | 0.03 | 0.03 | 0.03 |  | 0.02 | 0.03 | 0.03 |  | 0.02 | 0.03 | 0.03 | 0.00 | NS | NS | NS |
| 18:2 *c*9,*c*12 | 1.49 | 1.83 | 1.62 |  | 1.59 | 1.37 | 1.52 |  | 1.56 | 1.37 | 1.52 |  | 1.38 | 1.27 | 1.39 | 0.10 | NS | ** | * |
| 18:2 *t*12,*c*15 | 0.05 | 0.03 | 0.05 |  | 0.08 | 0.06 | 0.07 |  | 0.06 | 0.05 | 0.06 |  | 0.07 | 0.05 | 0.06 | 0.01 | ** | *** | NS |
| 20:0 | 0.14 | 0.17 | 0.14 |  | 0.14 | 0.15 | 0.13 |  | 0.13 | 0.13 | 0.13 |  | 0.13 | 0.13 | 0.12 | 0.01 | NS | ** | NS |
| 18:3 *t*9,*t*12,*c*15 | 0.01 | 0.01 | 0.01 |  | 0.00 | 0.00 | 0.00 |  | 0.00 | 0.00 | 0.00 |  | 0.00 | 0.00 | 0.00 | 0.00 | * | *** | *** |
| 18:3 *c*6,*c*9,*c*12 | 0.01 | 0.01 | 0.01 |  | 0.01 | 0.02 | 0.02 |  | 0.02 | 0.01 | 0.01 |  | 0.02 | 0.01 | 0.02 | 0.00 | NS | *** | NS |
| 20:1 *c*9 | 0.07 | 0.06 | 0.06 |  | 0.04 | 0.03 | 0.03 |  | 0.03 | 0.02 | 0.03 |  | 0.02 | 0.03 | 0.02 | 0.00 | ƚ | *** | NS |
| 20:1 *c*11 | 0.05 | 0.06 | 0.04 |  | 0.06 | 0.04 | 0.06 |  | 0.07 | 0.06 | 0.07 |  | 0.07 | 0.05 | 0.07 | 0.00 | * | *** | *** |
| 18:3 *c*9,*c*12,*c*15 | 0.54 | 0.40 | 0.70 |  | 0.38 | 0.33 | 0.40 |  | 0.38 | 0.32 | 0.37 |  | 0.42 | 0.35 | 0.38 | 0.04 | * | *** | ** |
| 18:2 *c*9,*t*11 | 0.54 | 0.41 | 0.57 |  | 0.50 | 0.33 | 0.46 |  | 0.43 | 0.33 | 0.40 |  | 0.44 | 0.33 | 0.39 | 0.03 | ** | *** | NS |
| 21:0 | 0.02 | 0.02 | 0.01 |  | 0.00 | 0.00 | 0.00 |  | 0.00 | 0.00 | 0.00 |  | 0.00 | 0.00 | 0.00 | 0.00 | * | *** | ** |
| 18:2 *t*11,*t*13 | 0.01 | 0.01 | 0.01 |  | 0.00 | 0.00 | 0.00 |  | 0.00 | 0.00 | 0.00 |  | 0.00 | 0.00 | 0.00 | 0.00 | NS | *** | NS |
| 18:2 *t*7,*t*9/*t*10,*t*12 | 0.02 | 0.02 | 0.02 |  | 0.02 | 0.02 | 0.02 |  | 0.02 | 0.02 | 0.02 |  | 0.02 | 0.02 | 0.02 | 0.00 | ƚ | NS | NS |
| 20:2 *c*11,*c*14 | 0.01 | 0.02 | 0.02 |  | 0.03 | 0.03 | 0.04 |  | 0.03 | 0.03 | 0.04 |  | 0.03 | 0.04 | 0.04 | 0.00 | NS | *** | NS |
| 22:0 | 0.05 | 0.05 | 0.05 |  | 0.06 | 0.06 | 0.05 |  | 0.05 | 0.06 | 0.06 |  | 0.06 | 0.06 | 0.06 | 0.00 | NS | * | NS |
| 20:3 *c*8,*c*11,*c*14 | 0.07 | 0.09 | 0.08 |  | 0.09 | 0.09 | 0.08 |  | 0.10 | 0.10 | 0.10 |  | 0.10 | 0.10 | 0.10 | 0.01 | NS | ** | NS |
| 22:1 *c*13 | 0.01 | 0.01 | 0.01 |  | 0.01 | 0.02 | 0.01 |  | 0.01 | 0.01 | 0.00 |  | 0.01 | 0.01 | 0.01 | 0.00 | NS | ** | NS |
| 20:3 *c*11,*c*14,*c*17 | 0.01 | 0.01 | 0.02 |  | 0.01 | 0.01 | 0.01 |  | 0.01 | 0.01 | 0.00 |  | 0.02 | 0.01 | 0.02 | 0.00 | NS | * | ƚ |
| 20:4 *c*5,*c*8,*c*11,*c*14 | 0.15 | 0.22 | 0.16 |  | 0.12 | 0.11 | 0.11 |  | 0.12 | 0.11 | 0.11 |  | 0.12 | 0.11 | 0.11 | 0.01 | NS | *** | * |
| 23:0 | 0.00 | 0.00 | 0.01 |  | 0.03 | 0.03 | 0.03 |  | 0.03 | 0.03 | 0.03 |  | 0.03 | 0.03 | 0.03 | 0.00 | NS | *** | NS |
| 20:5 *c*5,*c*8,*c*11,*c*14,*c*17 | 0.09 | 0.05 | 0.11 |  | 0.04 | 0.03 | 0.05 |  | 0.03 | 0.03 | 0.03 |  | 0.03 | 0.03 | 0.03 | 0.01 | ** | *** | ** |
| 24:0 | 0.03 | 0.04 | 0.04 |  | 0.04 | 0.04 | 0.04 |  | 0.04 | 0.04 | 0.05 |  | 0.04 | 0.05 | 0.04 | 0.00 | NS | NS | NS |
| 24:1 *c*15 | 0.01 | 0.01 | 0.01 |  | 0.03 | 0.02 | 0.03 |  | 0.00 | 0.00 | 0.00 |  | 0.01 | 0.00 | 0.01 | 0.00 | ƚ | *** | NS |
| 22:4 *c*7,*c*10,*c*13,*c*16 | 0.01 | 0.02 | 0.01 |  | 0.01 | 0.01 | 0.01 |  | 0.02 | 0.02 | 0.02 |  | 0.02 | 0.02 | 0.02 | 0.00 | NS | * | NS |
| 22:5 *c*7,*c*10,*c*13,*c*16,*c*19 | 0.16 | 0.12 | 0.17 |  | 0.09 | 0.07 | 0.10 |  | 0.07 | 0.05 | 0.07 |  | 0.06 | 0.04 | 0.06 | 0.01 | * | *** | NS |
| 22:6 *c*4,*c*7,*c*10,*c*13,*c*16,*c*19 | 0.03 | 0.02 | 0.03 |  | 0.00 | 0.00 | 0.00 |  | 0.00 | 0.00 | 0.00 |  | 0.00 | 0.00 | 0.00 | 0.00 | * | *** | * |
| Unknown | 0.55 | 0.43 | 0.58 |  | 0.68 | 0.59 | 0.59 |  | 0.45 | 0.44 | 0.48 |  | 0.51 | 0.50 | 0.46 | 0.03 | ** | *** | ** |
| *De novo* | 20.91 | 21.07 | 20.97 |  | 25.39 | 29.34 | 27.16 |  | 27.61 | 29.4 | 28.26 |  | 27.69 | 28.99 | 28.21 | 0.94 | NS | *** | NS |
| Mixed | 28.38 | 26.62 | 28.54 |  | 34.91 | 36.62 | 36.26 |  | 37.90 | 38.32 | 36.96 |  | 37.00 | 38.40 | 37.96 | 0.82 | NS | *** | NS |
| Preformed | 49.21 | 50.95 | 48.98 |  | 37.97 | 32.31 | 34.86 |  | 34.40 | 31.03 | 33.12 |  | 33.45 | 30.77 | 31.96 | 1.36 | NS | *** | NS |
| Total SFA*^b^* | 61.02 | 62.97 | 62.15 |  | 68.11 | 75.08 | 70.63 |  | 70.73 | 74.49 | 71.38 |  | 71.17 | 74.77 | 71.83 | 1.28 | * | *** | NS |
| Total MUFA*^c^* | 32.93 | 31.33 | 31.44 |  | 26.07 | 19.77 | 23.72 |  | 23.73 | 20.58 | 23.21 |  | 23.19 | 20.15 | 22.74 | 1.20 | * | *** | NS |
| Total PUFA | 3.65 | 3.54 | 4.08 |  | 3.50 | 2.92 | 3.38 |  | 3.29 | 2.81 | 3.17 |  | 3.22 | 2.77 | 3.04 | 0.18 | NS | *** | NS |
| Total n-6 FA*^d^* | 1.75 | 2.18 | 1.89 |  | 1.85 | 1.62 | 1.77 |  | 1.85 | 1.63 | 1.79 |  | 1.68 | 1.54 | 1.69 | 0.11 | NS | ** | ** |
| Total n-3 FA*^e^* | 0.83 | 0.60 | 1.02 |  | 0.53 | 0.44 | 0.56 |  | 0.49 | 0.41 | 0.47 |  | 0.54 | 0.42 | 0.49 | 0.05 | * | *** | ** |
| n-6:n-3 ratio | 2.19 | 3.75 | 1.98 |  | 3.51 | 3.69 | 3.23 |  | 3.78 | 4.03 | 3.79 |  | 3.15 | 3.74 | 3.47 | 0.19 | *** | *** | ** |
| Total CLA*^f^* | 0.57 | 0.44 | 0.60 |  | 0.52 | 0.35 | 0.48 |  | 0.45 | 0.35 | 0.41 |  | 0.46 | 0.35 | 0.41 | 0.03 | ** | *** | NS |
| Total 18:1 *t* | 2.54 | 2.42 | 2.86 |  | 2.19 | 1.96 | 2.01 |  | 1.81 | 1.68 | 1.72 |  | 1.71 | 1.54 | 1.51 | 0.12 | NS | *** | NS |
| Total branched chain FA | 1.76 | 1.68 | 1.68 |  | 1.50 | 1.51 | 1.53 |  | 1.63 | 1.55 | 1.59 |  | 1.74 | 1.69 | 1.79 | 0.07 | NS | *** | NS |
| Total *iso* FA | 0.97 | 0.92 | 0.97 |  | 0.87 | 0.92 | 0.92 |  | 0.91 | 0.94 | 0.93 |  | 1.06 | 1.06 | 1.11 | 0.04 | NS | *** | NS |
| Total *anteiso* FA | 0.79 | 0.76 | 0.71 |  | 0.63 | 0.59 | 0.61 |  | 0.72 | 0.62 | 0.66 |  | 0.68 | 0.63 | 0.68 | 0.03 | NS | *** | NS |
| Total odd-chain FA | 4.02 | 3.52 | 3.77 |  | 4.28 | 3.93 | 4.07 |  | 4.18 | 4.05 | 4.10 |  | 4.15 | 4.07 | 4.05 | 0.11 | ƚ | *** | NS |
